# Experimental Determination and Computational Prediction of Dehydroabietic Acid Solubility in (−)-α-Pinene + (−)-β-Caryophyllene + P-Cymene System

**DOI:** 10.3390/molecules27041220

**Published:** 2022-02-11

**Authors:** Yanmin Qin, Xiaopeng Chen, Linlin Wang, Xiaojie Wei, Weijian Nong, Xuejuan Wei, Jiezhen Liang

**Affiliations:** 1 Guangxi Key Laboratory of Petrochemical Resource Processing and Process Intensification Technology, School of Chemistry and Chemical Engineering, Guangxi University, Nanning 530004, China; qinyanmin19970112@163.com (Y.Q.); lilm@gxu.edu.cn (X.C.); wanglinlin1971@sina.com (L.W.); wxjgu@sina.com (X.W.); wei_xue_juan@163.com (X.W.); 2 China Academy of Science and Technology Development Guangxi Branch, Nanning 530022, China; weijiannong1208@163.com; 3 Guangxi Sci-Tech Development Forest-Like Technology Co., Ltd., Nanning 530022, China

**Keywords:** COSMO-RS, solubility, dehydroabietic acid

## Abstract

The solubility of dehydroabietic acid in (−)-α-pinene, p-cymene, (−)-β-caryophyllene, (−)-α-pinene + p-cymene, (−)-β-caryophyllene + p-cymene and (−)-α-pinene + (−)-β-caryophyllene were determined using the laser monitoring method at atmospheric pressure. The solubility of dehydroabietic acid was positively correlated with temperature from 295.15 to 339.46 K. (−)-α-pinene, p-cymene, and (−)-β-caryophyllene were found to be suitable for the solubilization of dehydroabietic acid. In addition, the non-random two liquid (NRTL), universal quasi-chemical (UNIQUAC), modified Apelblat, modified Wilson, modified Wilson–van’t Hoff, and λh models were applied to correlate the determined solubility data. The modified Apelblat model gave the minor deviation for dehydroabietic acid in monosolvents, while the λh equation showed the best result in the binary solvents. A comparative analysis of compatibility between solutes and solvents was carried out using Hansen solubility parameters. The thermodynamic functions of Δ_sol_H^0^, Δ_sol_S^0^, Δ_sol_G^0^ were calculated according to the van’t Hoff equation, indicating that the dissolution was an entropy-driven heat absorption process. The Conductor-like Screening Model for Real Solvents (COSMO-RS) combined with an experimental value was applied to predict the reasonable solubility data of dehydroabietic acid in the selected solvents systems. The interaction energy of the dehydroabietic acid with the solvent was analyzed by COSMO-RS.

## 1. Introduction

Pine resin is an inexpensive and biodegradable natural resource, which is abundant in pine and coniferous trees [1]. It is commonly used as an antimicrobial agent [2], paint, ink toner, and for coatings [3,4]. The most important products of pine resin are rosin and turpentine obtained via distillation [5]. Rosin is non-volatile in normal temperature since the boiling point is 250 °C at 0.66 kPa [6]. Rosin is composed mostly of rosin acids and some neutral matter [7]. The main components of rosin are abietic-type resin acid and primary-type resin acid. Turpentine is a terpene mixture mainly composed of monoterpenes such as (−)-α-pinene, β-pinene, camphene, p-cymene, and sesquiterpenes such as longifolene and (−)-β-caryophyllene. The monoterpenes are volatile components in a boiling point range of 155–175 °C at atmospheric pressure, while the boiling points of sesquiterpenes are in a range of 254–256 °C at atmospheric pressure.

About 1.3 million tons of pine resin is produced annually in China [8]. The collection of pine resin is the most significant and fundamental part of the rosin and turpentine industry chain. Resin tapping from live pine trees is normally performed every 10–15 days between April and October by the ‘debarking’ (bark-peeling) method [9]. Regular cuts are made in the trunk of the pine tree, allowing the pine resin to be secreted and collected in large quantities. The turpentine contained in the pine resin is continuously volatilized and lost in an open environment under the sun and rain, causing the turpentine content to decrease from around 30 to 15%. Due to the loss of turpentine, which can also act as a solvent for rosin acid, the rosin acid solidifies, thereby forming hard lumps that clog the resin tracts of the pine tree. Thus, the solid-liquid equilibrium of rosin acids and turpentine is essential to improve resin collection.

**Figure 1:**
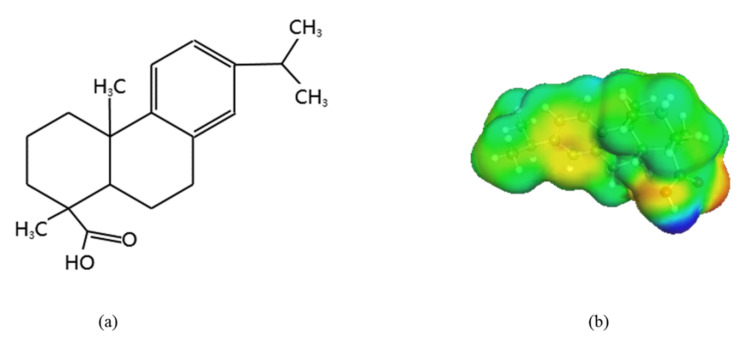
The molecular structure (**a**) and sigma surface (**b**) of dehydroabietic acid. The different colors shown in the sigma surface of benorilate have the following meanings: the red part represents the hydrogen acceptor region; the blue part represents the hydrogen donor region; and the green part represents the nonpolar region.

Dehydroabietic acid, one of the abietic-type resin acids contained in rosin, is a mono-carboxylic acid with a tricyclic phenanthrene skeleton [10,11,12], as shown in Figure 1. As can be seen, the molecular backbone of dehydroabietic acid is composed of a benzene ring rather than a double bond that is prone to oxidation. As a result, dehydroabietic acid is more stable than the other abietic-type resin acid. The unique structure of dehydroabietic acid offers a wide range of applications in the fields of pharmaceuticals, pesticides, surfactants, preparation of fine chemicals, and in the synthesis of biologically active substances [13].

Currently, the data on the solubility of dehydroabietic acid in solvents are mainly focused on alcohols [14] but have not yet been reported in the literature for turpentine systems. Therefore, this study aims to investigate the solid-liquid equilibrium of the rosin acids and turpentine. Dehydroabietic acid was applied as a model compound for rosin acid, while (−)-α-pinene, p-cymene and (−)-β-caryophyllene were employed as model compounds for turpentine. The solubility of dehydroabietic acid was predicted using the COSMO-RS model. The solubility of dehydroabietic acid in (−)-α-pinene, p-cymene, (−)-β-caryophyllene, (−)-α-pinene + p-cymene, (−)-α-pinene + (−)-β-caryophyllene, and p-cymene + (−)-β-caryophyllene were determined using a laser monitoring method. The corresponding molecular thermodynamic models of solid-liquid equilibrium were established, such as the non-random two liquid (NRTL), universal quasi-chemical (UNIQUAC), modified Apelblat, λh, modified Wilson, modified Wilson–van’t Hoff, etc. The compatibility of solute and solvent was described using the Hansen solubility parameter. The van’t Hoff equation was set to analyze the thermodynamic properties of the dissolution process.

## 2. Results and Discussion

### 2.1. Solid State Properties of Dehydroabietic Acid

**Figure 2:**
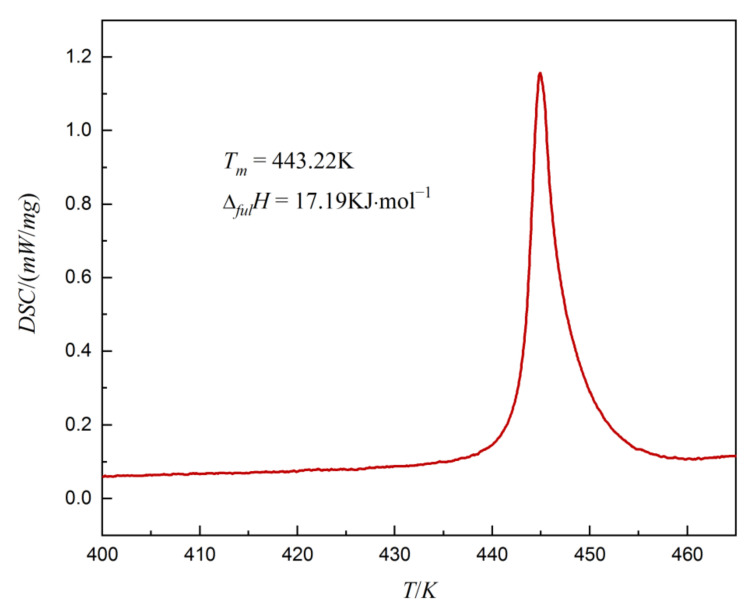
The differential scanning calorimetry (DSC) curve of dehydroabietic acid.

The differential scanning calorimetry (DSC) curve of dehydroabietic acid in its pure form is included in Figure 2. The onset melting temperature of dehydroabietic acid (*T_m_*) is 443.22 K, which is in general agreement with the literature values [15] (442.65 to 443.55 K in the literature). The melting enthalpy (Δ*_fus_H*) value for pure dehydroabietic acid is 17.19 kJ/mol as obtained by analyzing the DSC curve with the analysis software.

**Figure 3:**
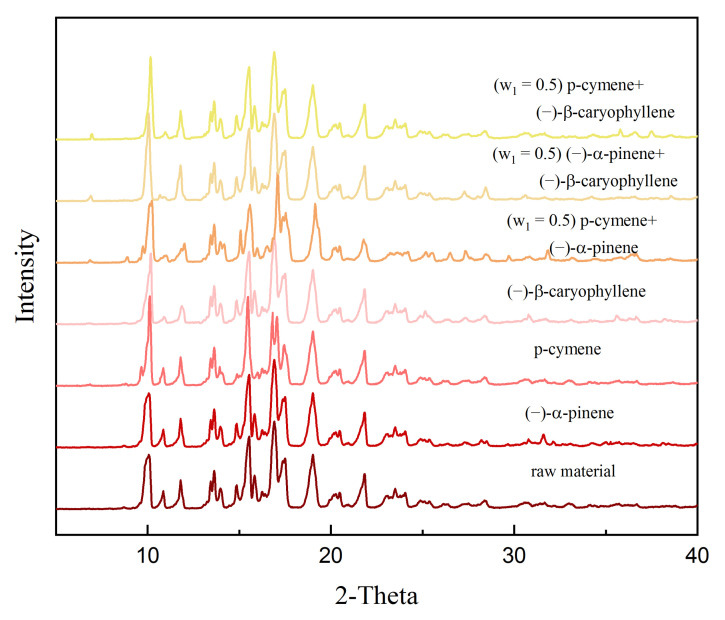
X-ray diffraction (XRD) patterns of raw dehydroabietic acid and recovered equilibrated dehydroabietic acid from three pure solvents and three binary mixed solvents.

**Figure 4:**
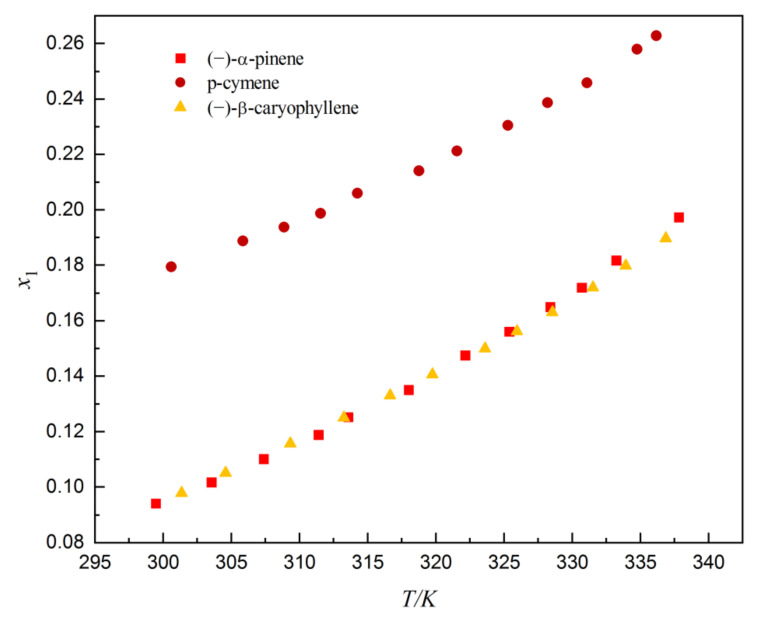
Mole fraction solubility of dehydroabietic acid in the three monosolvents.

The X-ray diffraction (XRD) patterns of raw dehydroabietic acid and recovered equilibrated dehydroabietic acid from three pure solvents and three binary mixed solvents are graphically shown in Figure 3. It can be seen from Figure 4 that the characteristic peaks of dehydroabietic acid in all of the solvent systems are the same as the raw material. This indicates that no crystal form transition occurred during the solid-liquid phase equilibrium of dehydroabietic acid in all pure and binary mixed solvent systems.

### 2.2. Experimental Solubility Data of Dehydroabietic Acid

**Figure 5:**
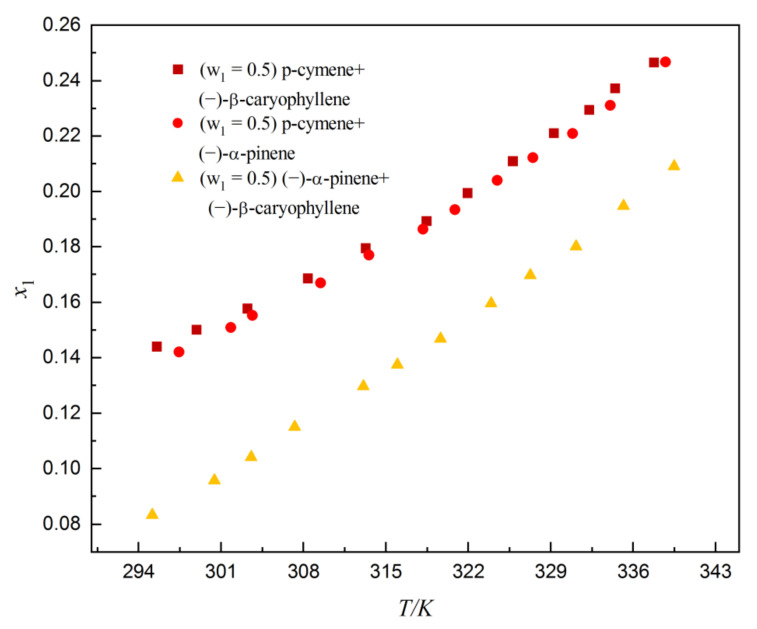
Mole fraction solubility of dehydroabietic acid in the three mixed solvents.

The solubility of dehydroabietic acid in (−)-α-pinene, p-cymene, and (−)-β-caryophyllene are shown in Table 1 and graphically plotted in Figure 4. It can be obviously observed that as the temperature increases from 299.45 to 337.85 K, the solubility of dehydroabietic acid increases. The solubility of dehydroabietic acid in p-cymene is significantly greater than that in (−)-α-pinene and (−)-β-caryophyllene; it may be that the polarity of p-cymene is greater than ethyl acetate (Table 2), which affects the interaction between the solute and the solvent. It can also be seen from Table 2 that the Δδ value was lower in (−)-α-pinene (Δδ_t_ = 3.1075 MPa^0.5^), p-cymene (Δδ_t_ = 2.6628 MPa^0.5^), and (−)-β-caryophyllene (Δδ_t_ = 2.9681 MPa^0.5^), indicating the complete solubilization of dehydroabietic acid in all these solvents according to this theory [16]. Overall, (−)-α-pinene, p-cymene, and (−)-β-caryophyllene were found to be suitable for the solubilization of dehydroabietic acid due to the close value of different HSPs of dehydroabietic acid with those of (−)-α-pinene, p-cymene, and (−)-β-caryophyllene. Table 3 and Figure 5 show the solubility of dehydroabietic acid in (−)-α-pinene + p-cymene, (−)-α-pinene + (−)-β-caryophyllene, and p-cymene + (−)-β-caryophyllene. The solubility of dehydroabietic acid in binary solvents containing p-cymene is significantly higher than that of free p-cymene.

### 2.3. Solubility Correlation

A regression analysis of the solubility of dehydroabietic acid in the three monosolvents was carried out with four thermodynamic models (modified Apelblat, λh, NRTL and UNIQUAC models). Three thermodynamic models (λh, modified Wilson and van’t-Hoff-Wilson) were employed to regress the solubility of dehydroabietic acid in the three binary solvents. The regression parameters for six models are presented in Table 4, Table 5, Table 6, Table 7, Table 8, Table 9 and Table 10. The reliability and suitability of the regression results were evaluated by using root mean square deviation (RMSD), relative deviation (RD) and average relative deviation (ARD) [18,19]. The mathematical expressions for RD, ARD and RMSD are the Equations (1)–(3), respectively.

(1) $$\mathrm{RD}=\frac{x_{1}^{exp}-x_{1}^{cal}}{x_{1}^{exp}}$$

(2) $$\mathrm{ARD}=\frac{\sum_{i=1}^{n}\left|\frac{x_{1}^{exp}-x_{1}^{cal}}{x_{1}^{exp}}\right|}{n}$$

(3) $$\mathrm{RMSD}={\left(\frac{\sum_{i=1}^{n}{\left(x_{1}^{exp}-x_{1}^{cal}\right)}^{2}}{n}\right)}^{1/2}$$

The RD, ARD and RMSD values are also presented in Table 4, Table 5, Table 6, Table 7, Table 8, Table 9, Table 10 and Tables S1 and S2. The relative deviations of the solubility of dehydroabietic acid in the three monosolvents are well distributed by using the four models. However, when the NRTL model is employed to correlate p-cymene, there is a distinct difference in the relative deviation distribution. The maximum RMSD for the mixture of p-cymene is 12.00745 in the NRTL model, and the maximum value of ARD is 4.89582%. The modified Apelblat model is applied to correlate the solubility of dehydroabietic acid in (−)-α-pinene, p-cymene and (−)-β-caryophyllene with the smallest average relative deviation of the four models. The calculated results, as shown in Table 4, indicate that the values of ARD are 0.29696, 0.185545, and 0.36719%, respectively. Similarly, the 10^3^RMSD has minimal values, which are 0.49179, 0.53532, and 0.61823. The modified Apelblat model equation offers excellent correlation results between the solubility of dehydroabietic acid in pure solvents and binary solvent mixtures in comparison to other model equations.

As can be seen from Table 8, Table 9 and Table 10, the average relative deviation obtained from the Wilson–van’t Hoff model for the solubility of dehydroabietic acid in (−)-β-caryophyllene + p-cymene is lower than that obtained from the Wilson model alone, indicating that the combined model is more effective in correlating the solubility of dehydroabietic acid in (−)-β-caryophyllene + p-cymene. As shown in Table S2, during the three models applied to correlate the determined solubility data of dehydroabietic acid in (−)-β-caryophyllene + p-cymene, the relative deviations of the other models are unevenly distributed, except for the λh model.

For the three models that correlated the solubility of dehydroabietic acid in p-cymene and (−)-α-pinene, the Wilson model alone showed an uneven distribution of relative deviations. However, for the binary solvents of p-cymene + (−)-α-pinene, the deviations of all models are unevenly distributed, with a particularly large gap in the Wilson model. The maximum RMSD for the binary solvents of (−)-α-pinene + (−)-β-caryophyllene is 5.41597 in the modified Wilson model, and the maximum value of ARD is 3.56843%.

Combining RD, ARD and RMSD, the solubility of dehydroabietic acid in binary solvents correlated with the modified Apelblat model and supplied superior results. The solubility of dehydroabietic acid in (−)-β-caryophyllene + p-cymene and (−)-α-pinene + p-cymene is appropriately correlated with the λh model. The solubility of dehydroabietic acid in (−)-α-pinene + (−)-β-caryophyllene correlated the most closely with the Wilson–van’t Hoff model.

### 2.4. Evaluation of Thermodynamic Models

In order to select the best correlation model for dehydroabietic acid in different solvents, the Akaike Information Criterion (AIC) [20] was employed. The AIC is expressed as follows:

(4) $$\mathrm{AIC}=-2\mathrm{lnL}\left(\theta \right)+2k$$

The L(*θ*) and *k* denotes the maximum likelihood value of the model, and the amount of estimable parameters to assess the model, respectively. AIC can also be expressed as:

(5) $$\mathrm{AIC}=\mathrm{Nln}\left(RSS/N\right)+2k$$

(6) $$\mathrm{RSS}=\overset{N}{\underset{i=1}{\sum }}{\left(x_{i}-x_{ci}\right)}^{2}$$

where *N* is the number of observations; RSS is the residual sum of squares; and *x_i_* and *x_ci_* are the experimental and calculated values of solute solubility, respectively. The AIC calculation results of each model are displayed in Table 11.

The Akaike weights are used to describe the results of the application of the model more clearly and are expressed as follows [21]:

(7) $$\omega_{i}=\frac{exp\left(\left(AIC_{min}-AIC_{i}\right)/2\right)}{\sum_{i=1}^{M}exp\left(\left(AIC_{min}-AIC_{i}\right)/2\right)}$$

where *M* is the amount of chosen models; *AIC_min_* is the lowest *AIC* value of the chosen models; and *AIC_i_* is the *AIC* value of the *i*th model.

The results of comparing the model correlations are shown in Table 11. In principle, the model with the lowest AIC value is the most appropriate model. It can be seen from Table 4 that, for the monosolvents, the lowest AIC values were obtained when correlating the solubility of dehydroabietic acid in (−)-α-pinene (−176.8191), and p-cymene (−174.7835) with the modified Apelblat. Moreover, the λh model (AIC = −171.9114) was the most appropriate model for describing the solubility of dehydroabietic acid in (−)-β-caryophyllene. For the binary solvents, the λh model could give the best performance for correlating the solubility of dehydroabietic acid in (−)-α-pinene + p-cymene (AIC = −167.0801) and (−)-β-caryophyllene + p-cymene (AIC = −152.2803), while the modified Wilson with van’t Hoff model was the best model for (−)-α-pinene + (−)-β-caryophyllene (AIC = −143.3940).

### 2.5. Thermodynamic Properties of the Solution

The thermodynamic analysis is contributed in order to understand the dissolution process of dehydroabietic acid. The relationship between solubility and the thermodynamic parameters of dissolution is derived from the van’t Hoff equation [22] and expressed as follows.

(8) $${\left(\frac{\partial lnx_{1}}{\partial \left(1/T-1/T_{hm}\right)}\right)}_{p}=-\frac{\Delta_{sol}H^{0}}{R}$$

(9) $$T_{hm}=\frac{n}{\sum_{j}^{n}=\frac{1}{T_{i}}}$$

where *T_i_* denotes the experimental temperature; *n* denotes the number of temperature points; and *x*_1_ means the mole fraction solubility of dehydroabietic acid [23]. The fitted curve *lnx*_1_ (1/*T* − 1/*T_hm_*) with a slope value of enthalpy of solution ($\Delta_{sol}H^{0}$), entropy of solution ($\Delta_{sol}S^{0}$), and Gibbs free energy of solution ($\Delta_{sol}G^{0}$), can be calculated according to the following equation [24]:

(10) $$\Delta_{sol}G^{0}=-R\times T_{hm}\times Intercept$$

(11) $$\Delta_{sol}S^{0}=\frac{\Delta_{sol}H^{0}-\Delta_{sol}G^{0}}{T_{hm}}$$

The Gibbs free energy was affected by enthalpy and entropy changes. In order to further understand how the thermodynamic parameters affect the Gibbs free energy, it is necessary to introduce the concept of the thermodynamic weighting coefficients $\delta_{H}$ and $\delta_{TS}$ [25,26], which can be defined as follows:

(12) $$\delta_{H}=\frac{\left|\Delta_{sol}H^{0}\right|}{\left|\Delta_{sol}H^{0}\right|\;+\;\left|T_{hm}\Delta_{sol}S^{0}\right|}$$

(13) $$\delta_{TS}=\frac{\left|T_{hm}\Delta_{sol}S^{0}\right|}{\left|\Delta_{sol}H^{0}\right|\;+\;\left|T_{hm}\Delta_{sol}S^{0}\right|}$$

**Figure 6:**
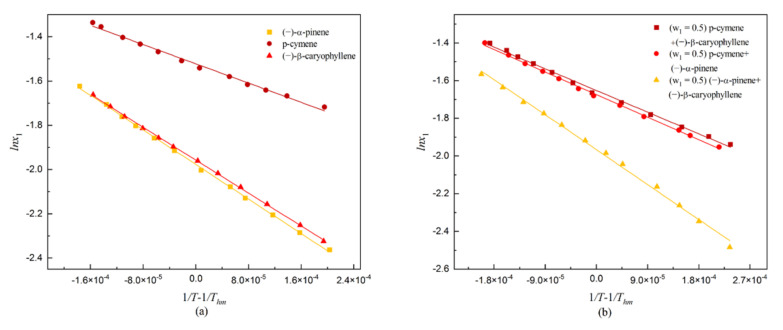
Plot of ln*x*_1_ against 1/*T* − 1/*T_hm_* for dehydroabietic acid in selected solvents: (**a**) indicates in monosolvents; (**b**) indicates in mixed solvents.

The values of Δ_sol_G^0^, Δ_sol_H^0^, Δ_sol_S^0^, δ_H_ and δ_TS_ of dehydroabietic acid in solvents were calculated according to Equations (8)–(13). The results are shown in Table 12. The values of intercept and slope were obtained via regression of the experimental solubility data. The curves of *lnx*_1_ versus (1/*T*−1/*T_hm_*) for dehydroabietic acid in three monosolvents and three binary mixed solvents are shown in Figure 6.

It can be seen from Table 12 that Δ_sol_H^0^ is positive in all cases, suggesting that the process of dissolution of dehydroabietic acid is endothermic, which is favorable for further dissolution. Thus, the solubility becomes greater as the temperature increases, which is consistent with the solubility results. It also appears that the compatibility of dehydroabietic acid with monosolvents is basically ranked in the order of (−)-α-pinene < (−)-β-caryophyllene < p-cymene. The order of compatibility of dehydroabietic acid with binary solvents is (−)-α-pinene + (−)-β-caryophyllene < (−)-α-pinene + p-cymene < p-cymene + (−)-β-caryophyllene.

The order of Δ_sol_G^0^ of dehydroabietic acid in monosolvents is (−)-α-pinene > (−)-β-caryophyllene > p-cymene. The order of Δ_sol_G^0^ of dehydroabietic acid in binary solvents is (−)-α-pinene + (−)-β-caryophyllene > (−)-α-pinene + p-cymene > p-cymene + (−)-β-caryophyllene. This indicates that the larger the value of Δ_sol_G^0^, the smaller the solubility. Conversely, the largest δ_H_ for dehydroabietic acid in monosolvents is p-cymene, and the maximum δ_H_ in binary solvents is p-cymene + (−)-β-caryophyllene. In addition, the value of entropy Δ_sol_S^0^ is also positive, indicating that the dissolution process of dehydroabietic acid is irreversible. Moreover, in the dehydroabietic acid dissolution processes, δ_H_ is always greater than that of δ_TS_, which shows that enthalpy is the main contributor to the Gibbs free energy change.

### 2.6. Solubility Prediction

**Figure 7:**
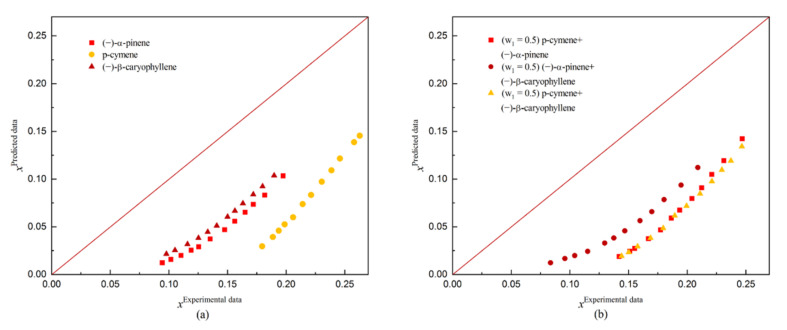
Comparison of COSMO-RS predicted and experimental solubility of dehydroabietic acid: (**a**) indicates the comparison in monosolvents; and (**b**) indicates the comparison in binary solvents.

**Figure 8:**
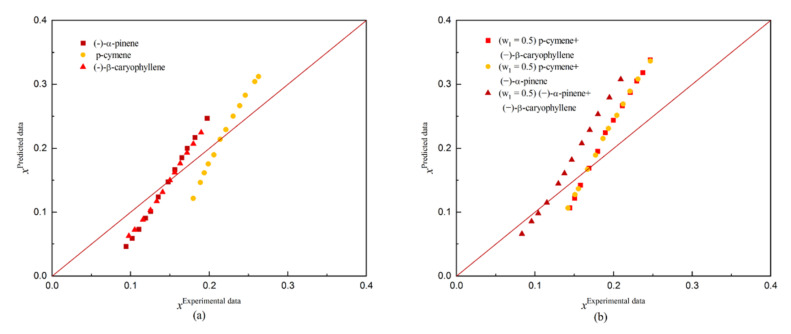
Comparison of COSMO-RS predicted and experimental solubility of dehydroabietic acid: (**a**) indicates the comparison in monosolvents; and (**b**) indicates the comparison in binary solvents.

Conductor-like Screening Model for Real Solvents (COSMO-RS) predicts the thermodynamic properties of any mixed solution based on a quantitative calculation without any experimental data or group interaction parameters. The solubility of dehydroabietic acid in three monosolvents ((−)-α-pinene, p-cymene, and (−)-β-caryophyllene) and three binary solvents was predicted by COSMO-RS. The predicted results are shown in Figure 7 and Figure 8 along with the comparison between the predicted and experimental solubility.

During the fitting of solubility for dehydroabietic acid in organic solvents by using the COSMO-RS model, the results obtained were unsatisfactory, with ARD values above 60% (the largest ARD value reaching 69.31549%) in each group when only the experimental ΔG_fus_ (T_m_ = 443.22 K, ΔH_fus_ =19.17 kJ/mol) is used as the conditioning parameter of COSMO-RS. However, the best fit was obtained when using experimental reference solubility data (x_i_), with the largest ARD value of 23.75923% in several sets of predictions. This may be attributed to the ΔG_fus_ of the solid solutes, as this was estimated using quantitative structural property correlation methods. In contrast, the ΔG_fus_ parameters obtained during the estimation process were only calculated quantitatively and were not validated by experimental data. As a result, the estimated values deviate significantly from the experimental values.

**Figure 9:**
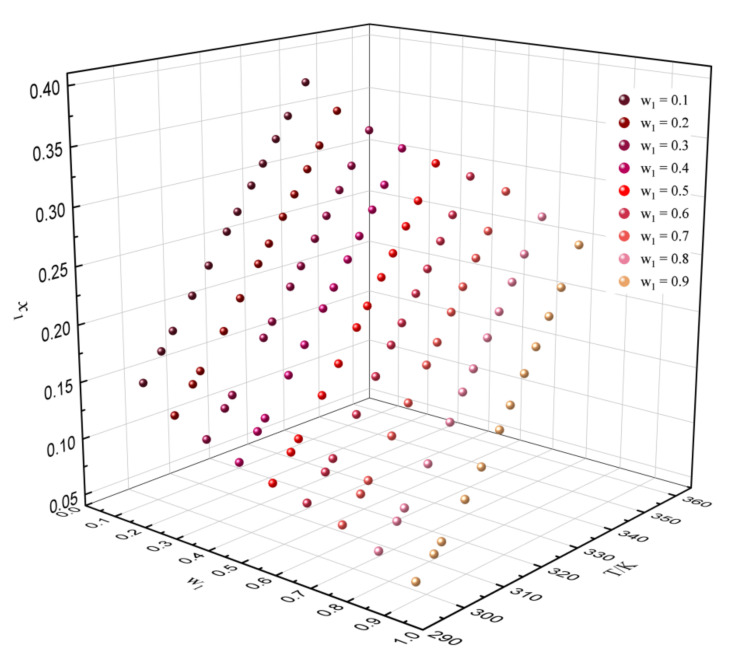
Solubility prediction data of dehydroabietic acid in the binary solvent of (−)-α-pinene (w_1_) + p-cymene with various mass fractions at different temperatures.

**Figure 10:**
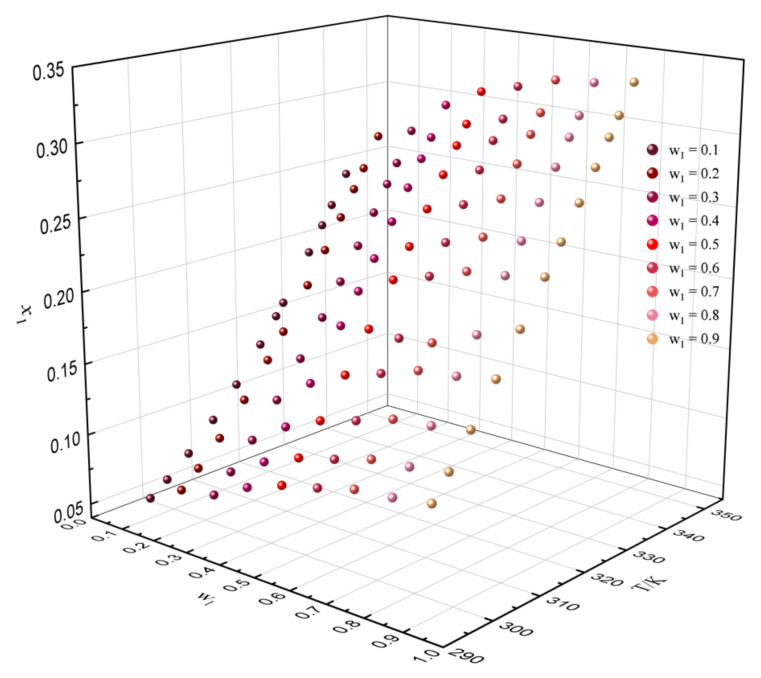
Solubility prediction data of dehydroabietic acid in the binary solvent of p-cymene (w_1_) + (−)-β-caryophyllene with various mass fractions at different temperatures.

**Figure 11:**
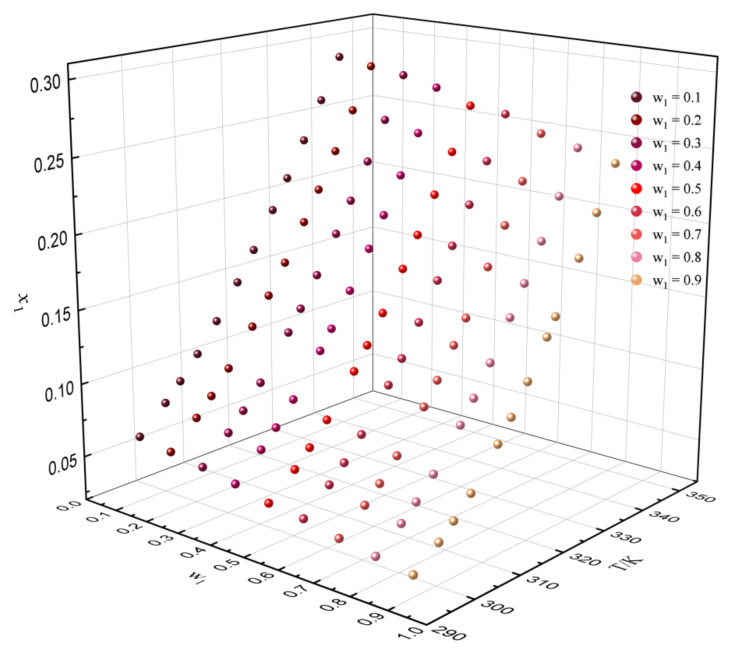
Solubility prediction data of dehydroabietic acid in the binary solvent of (−)-α-pinene (w_1_) + (−)-β-caryophyllene with various mass fractions at different temperatures.

It can be concluded that the method of predicting solubility with experimental reference solubility data is more reliable. Thus, the experimental data in Table 13 were used to predict the solubility of dehydroabietic acid in binary mixtures with various mass fractions at different temperatures. The predicted results are shown in Figure 9, Figure 10 and Figure 11. In the binary solvents containing p-cymene, it can be clearly found that the solubility of dehydroabietic acid increased with the increasing temperature and mole fraction of p-cymene. Moreover, in (−)-α-pinene + (−)-β-caryophyllene, the solubility of dehydroabietic acid increased with the increasing temperature and decreasing mole fraction of (−)-α-pinene.

### 2.7. Molecular Interaction Energies Analysis

**Figure 12:**
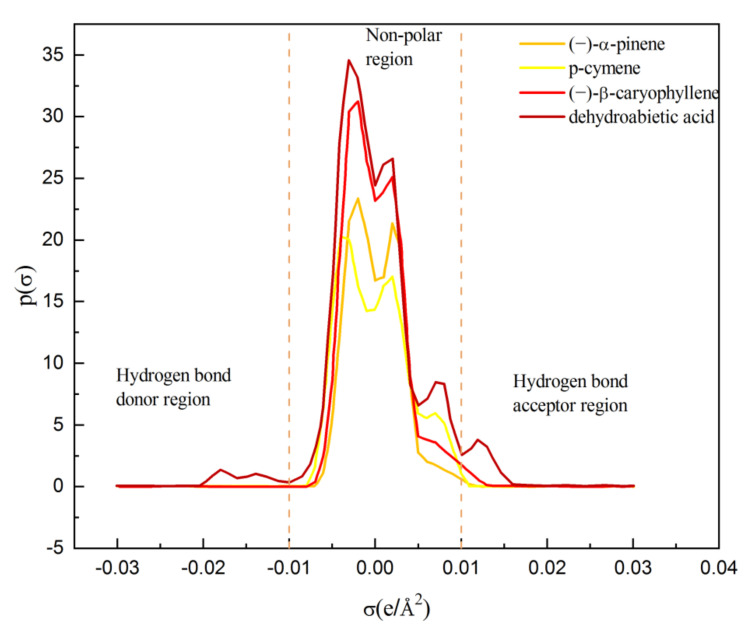
Sigma profiles of dehydroabietic acid and solvents.

The σ-profiles can be used to describe the salvation and intermolecular interactions between dehydroabietic acid and the solvents. The shielding charge density distribution (σ-profile) on the molecular surface was calculated by the TURBOMOLE software package under the BP (B88-VWN-P86) and TZVP basis sets. The σ-profiles of the solvent and solute were calculated as shown in Figure 12. As can be seen from the figure, (−)-α-pinene is the least polar compound, which is reflected in the narrow distribution of charge density around zero. In the −0.01 to −0.02 hydrogen bond donor region, two broad peaks are generated by hydroxyl oxygen for dehydroabietic acid. One broad peak is generated by carbonyl in the hydrogen bond acceptor region of 0.01 to 0.02, reflecting the fact that dehydroabietic acid is the most polar compound with both a hydrogen bond donor and a hydrogen bond acceptor. Further, in the non-polar region of −0.01 to 0.01, both p-cymene and dehydroabietic acid have a similar three peaks, as they both share the non-polar group benzene ring.

COSMO-RS calculations show that the hydrogen bond donor moment and the hydrogen bond acceptor moment of the dehydroabietic acid molecule are 2.8643 and 1.1416, respectively, indicating that the acidity of the hydrogen bond in dehydroabietic acid is greater than the basicity. Therefore, dehydroabietic acid is more likely to be solvated with a strong hydrogen basic solvent. The hydrogen bond acceptor moments of (−)-α-pinene and (−)-β-caryophyllene were 0.0008 and 0.0345, respectively. There is a more obvious stretching peak in the hydrogen bond acceptor region of the graph for (−)-β-caryophyllene. In contrast, the hydrogen bond acceptor moment for the highly soluble p-cymene is 0. However, the solubility of dehydroabietic acid in the p-cymene is the strongest. It shows that the acidity and basicity of the non-polar solvent has little influence on the solubility of dehydroabietic acid.

**Figure 13:**
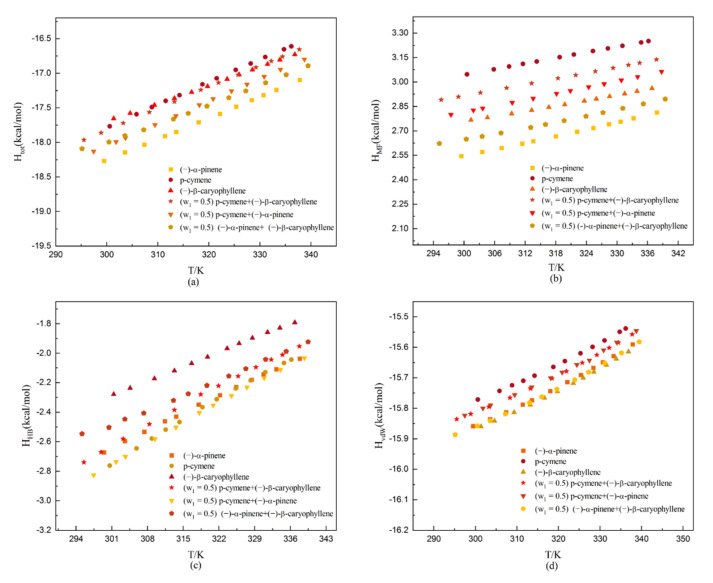
The molecular interaction energies of dehydroabietic acid in the studied solvents: (**a**) total mean interaction energy (H_tot_); (**b**) misfit interaction energy (H_MF_); (**c**) hydrogen-bond interaction energy (H_HB_); and (**d**) van der Waals interaction energy (H_vdW_).

The different interaction energies between the dehydroabietic acid molecules and the solvent molecules have implications for the study of their solubility [27]. The COSMOtherm program can describe the intermolecular interaction forces in terms of molecular surface interactions. When performing solubility calculations with COSMO-RS, the interaction forces between the solvent and the solute were obtained; the results are shown in Figure 13. In this paper, the misfit interaction energy (H_MF_), hydrogen-bond interaction energy (H_HB_), van der Waals interaction energy (H_vdW_), and total mean interaction energy (H_tot_) of dehydroabietic acid in solvents were calculated using COSMO-RS.

In general, the more positive the misfit interaction energy, the weaker the electrostatic interaction was between molecules [28]. As can be seen from Figure 13b, dehydroabietic acid has the weakest electrostatic interaction with p-cymene during dissolution in monosolvents and the weakest electrostatic interaction with p-cymene + (−)-β-caryophyllene in binary solvents. This trend is the opposite to the experimental solubility. The dissolution process is complicated, and the influencing factors may include van der Waals force, the degree of molecular association caused by the polarity of the molecule, the electrostatic interaction, solvation, the relative molecular mass of the solvent and solute, and the type and number of dissolving active groups, etc. The reason for this phenomenon of anomaly can be attributed to the weak influence of electrostatic effects on the studied system.

In addition, the more negative the hydrogen bonding interaction energy, the stronger the intermolecular hydrogen bonding [29]. As shown in Figure 13c, the strongest intermolecular hydrogen bonding is obtained with p-cymene during the dissolution in monosolvents. After the increase in temperature to 322 K, the values of the hydrogen bonding interaction energies of dehydroabietic acid in (−)-α-pinene and p-cymene are close to each other. Therefore, the strongest intermolecular hydrogen bonding in binary solvents is (−)-α-pinene + p-cymene. This suggests that hydrogen bonding interactions have a certain influence on the dissolution of dehydroabietic acid.

The van der Waals force is an attractive force present between molecules and is much weaker than chemical bonds. The higher the van der Waals force is, the higher the melting and boiling points of substances are. For substances with similar composition and structure, the van der Waals force increases with the increase in relative molecular mass. As can be seen from Figure 13d, the van der Waals interaction energy of (−)-β-caryophyllene is the strongest during the dissolution of dehydroabietic acid in monosolvents. Correspondingly, its boiling point and relative molecular mass are also the largest. In binary solvents, the van der Waals interaction energy of (−)-α-pinene + (−)-β-caryophyllene is the strongest.

As can be seen from Figure 13a, the total average interaction energy for the dissolution of dehydroabietic acid in the studied solvent is negative. It suggests that the dissolution process is thermodynamically favorable.

## 3. Experimental

### 3.1. Materials

Disproportionated rosin was provided from Wuzhou Sun Shine Forestry and Chemicals Co., Ltd. of Wuzhou, China. (−)-α-pinene (mass fraction purity > 0.98) was provided from Aladdin Biochemical Co., Ltd. of Shanghai, China. The p-cymene (mass fraction purity > 0.99) and (−)-β-caryophyllene (mass fraction purity > 0.99) were supplied by Adamas Pharmaceuticals, Inc and TCI (Shanghai, China) Development Co., Ltd. Pharmaceuticals, Inc, respectively.

The acute toxicity was LD > 48 mg/kg (rat abdominal cavity) for (−)-β-caryophyllene, and LD50 of 3700 mg/kg (oral in rats) for (−)-α-pinene. P-cymene is an irritant drug; irritating the eyes, respiratory system, and skin.

Further descriptions of the chemicals involved in this work can be found in Table 14.

### 3.2. Purification of Dehydroabietic Acid

The dehydroabietic acid was prepared with disproportionated rosin as raw material using the method reported in Ref. [30]. The disproportionated rosin was completely dissolved using 95% ethanol and then put into a stirred reactor. The disproportionated rosin was reacted with 2-ethanolamine at 35 °C for 50 min by means of a reaction–crystallization coupled with an ultrasonic wave to obtain 2-aminoethanol salt. After the reaction, the 2-aminoethanol salt of dehydroabietic acid crystals were obtained via vacuum filtration. The 2-aminoethanol salt of dehydroabietic acid crystals was dissolved with 50% ethanol and then extracted 3 times by adding isooctane to a water bath at 70 °C. Subsequently, a pure 2-aminoethanol salt of dehydroabietic acid was obtained and recrystallized 3 times from 50% ethanol. Finally, pure dehydroabietic acid with a purity of 99% was obtained via acidification with dilute hydrochloric acid. The specific rotation was +62.0°.

The mass fraction of the materials mentioned above were analyzed using gas chromatography. Dehydroabietic acid contains carboxyl groups and is a polar substance with a high boiling point; therefore, it needs to be pre-treated by adding a 6% tetramethylammonium hydroxide methanol solution before it can be injected into the chromatograph. First, a small amount of the sample was dissolved in methanol. Then 1–2 drops of phenolphthalein indicator and then 6% tetramethylammonium hydroxide methanol solution were added until the solution was pink, which did not fade after gentle shaking. Finally, the sample was injected for analysis. The samples were analyzed by an Agilent 7820A gas chromatograph system equipped with a DB-5MS capillary column (30 m × 320 μm × 0.25 μm) and a flame ionization detector (FID). N_2_ of 99.999% purity was used as the carrier gas, at a constant flow rate of 25 mL·min^−1^. The injector temperature was 523.15 K and the detector temperature was 563.15 K. The sample volume was 0.4 μL. The column temperature was 373.15 K at the beginning, ramped to 543.15 K at 5 K·min^−1^, and finally held at 543.15 K for 5 min. The mass fraction of 99% for dehydroabietic acid was obtained using gas chromatography (GC) analysis.

### 3.3. Characterization of the Solid Phase

The melting point T_m_ and melting enthalpy Δ_fus_H of dehydroabietic acid were determined using differential scanning calorimetry (NETZSCH STA 449F3, Nanining, China). The differential scanning calorimetry (DSC) instrument was first pre-calibrated based on pure indium. Subsequently, 3.31 mg of dehydroabietic acid was tested at a ramp rate of 5 K-min^−1^ in the range of 30–300 °C under nitrogen protection at a gas flow rate of 40 mL/min.

It is necessary to determine whether the selected solvents changed the crystal morphology of dehydroabietic acid. The crystal structure of dehydroabietic acid in pure form and when recovered from the solvent was determined by X-ray diffraction (XRD). The samples were scanned with Cu-Kα radioactivity. The XRD patterns of the sample were conducted at a scanning speed of 6°/min from 5 to 40°.

### 3.4. Reliability Analysis of Devices and Methods

Solubility is the basic thermodynamic data for chemical processes such as crystallization and separation as well as product purification. It requires a high degree of accuracy and, therefore, the reliability of the experimental methods and apparatus used for the determination of solubility needs to be checked. The solubility of potassium chloride and benzoic acid in water has been widely reported. Thus, the organic benzoic acid-water and the inorganic potassium chloride-water systems were selected as standard systems for this paper. The solubility of benzoic acid and potassium chloride in water was determined using a laser monitoring method and compared with literature values.

**Figure 14:**
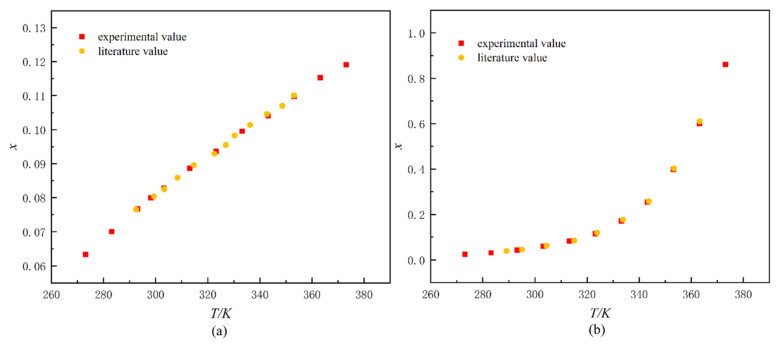
The solubility of potassium chloride in water (**a**) and the solubility of benzoic acid in water (**b**).

As can be seen in the Figure 14, the data on the solubility of benzoic acid and potassium chloride in water determined using the laser monitoring method matches the literature data [31,32]. The results show that the experimental equipment and methods used to determine solubility in this paper are reliable.

### 3.5. Solubility Measurements

The solubility of dehydroabietic acid in three monosolvents: ((−)-α-pinene, p-cymene, and (−)-β-caryophyllene), and three binary solvents: (p-cymene + (−)-β-caryophyllene, (−)-α-pinene + (−)-β-caryophyllene, and p-cymene + (−)-α-pinene) were measured using the laser monitoring method at P = 101.3 kPa. The device used in the laser monitoring method consists of a jacketed glass vessel (60 cm^3^), a temperature controlling system, a laser monitoring system, and a stirring system. The jacketed glass vessel could be maintained at the desired temperature within ± 0.1 K by circulating water in a cryogenic thermostat (HS-205, Beijing, China). The laser monitoring system was a combination of a laser generator, photoelectric transformer, and a light strength display. The jacketed glass vessel and the laser monitoring system were placed in a black box to prevent the laser intensity from the light outside, improving the stability and accuracy of the experiment. In the experiments, a predetermined amount of the dehydroabietic acid and the solvent were precisely weighed with an electronic analytical balance (Mettler Toledo AB135-S, Changshu, China, accuracy: 0.0001 g) and then added to the dissolution kettle. The magnetic stirrer was then turned on to mix the solid and liquid phases. The cryogenic thermostat was also turned on, and the temperature was raised approximately 1 K·h^−1^. The laser beam from the laser generator entered the dissolution kettle from one side and was accepted by the photoelectric transformer on the other side. The laser signals were converted into electrical signals and directed by the light strength display. In the early stages of the dissolution, most of the solid solutes of dehydroabietic acid were not dissolved but suspended in the liquid, which made most or even all of the incident laser light to become reflected and obscured. Hence, the value on the light intensity display was meager. With the temperature being slowly increased (less than 0.2 K/h near the equilibrium temperature), the solid particles gradually dissolved into the liquid phase, while the value on the light intensity display gradually increased. The laser intensity increased to the maximum value and tended to stabilize when the last dehydroabietic acid particle of the solute was fully dissolved, and the laser intensity reached the maximum. The temperature that corresponded to the maximum intensity was recorded as the equilibrium temperature.

The mole fraction solubility of dehydroabietic acid (x_1_) in the selected solvent is calculated by Equations (14) and (15).

(14) $$x_{1}=\frac{m_{1}/M_{1}}{m_{1}/M_{1}+m_{2}/M_{2}}$$

(15) $$x_{1}=\frac{m_{1}/M_{1}}{m_{1}/M_{1}+m_{2}/M_{2}+m_{3}/M_{3}}$$

where $m_{1}$ indicates the mass of dehydroabietic acid; $m_{2}$ and $m_{3}$ indicate the mass of the solvents; $M_{1}$ represents the molar mass of the dehydroabietic acid; $M_{2}$ and $M_{3}$ represents the molar mass of the solvent; and x_1_ denotes a molar fraction of the dehydroabietic acid in the solvent mixture.

The mass fraction of the solvent is expressed by the following equation:

(16) $$w_{1}=\frac{m_{1}}{m_{1}+m_{2}}$$

where $m_{1}$ and $m_{2}$ denote the mass of solvent 1 and solvent 2, respectively.

## 4. Theoretical Basis

### 4.1. Hansen Solubility Parameter (HSP)

The Hansen total solubility parameter (HSP) consists of a dispersion component (*E_D_*), a dipole component (*E_P_*), a hydrogen bonding component (*E_H_*), and the total solubility parameter of the molecular structure, which can be calculated by using the principle of summation.

(17) $$E=E_{D}+E_{P}+E_{H}$$

Dividing it by the molar volume gives the square of the total solubility parameter (δ^2^) [17,33].

(18) $$E/V=E_{D}/V+E_{P}/V+E_{H}/V$$

(19) $$\delta^{2}=\delta_{D}^{2}+\delta_{P}^{2}+\delta_{H}^{2}$$

The “similarity and mutual solubility” theory was used to evaluate the variability of the HSP (Δ*δ_t_*) [34], which is expressed as Equation (20).

(20) $$\Delta \delta_{t}=\left|\delta_{t2}-\delta_{t1}\right|$$

where *δ_t_*_1_ and *δ_t_*_2_ are the total HSP of solute and solvent, respectively. The HSPs of dehydroabietic acid and its solvent are shown in Table 15 and Table S3.

### 4.2. The Modified Apelblat Model

The modified Apelblat equation, which ignores the effect of the solute activity coefficient at atmospheric pressure, has a simpler form with three correlation parameters [35] (*A*, *B*, *C*) (See Table 4). This method is suitable for the interpolation correlation of solubility data and fits the experimental data well. However, for systems with a wide range of solubility values, the correlation results of the modified Apelblat equation are biased. The formula is given as follows:

(21) $$lnx=A+\frac{B}{T}+Cln\left(T\right)$$

This empirical equation describes the relationship between solubility and temperature, in which *x*_1_ is the molar fraction of dehydroabietic acid dissolved in the solvents at the absolute temperature *T*. However, for systems with a wide range of solubility values, the correlation results of the Apelblat equation deviate considerably.

### 4.3. λh Model

The λh equation is a semi-empirical equation of the following Equation (22) [36,37]:

(22) $$ln\left[1+\frac{\lambda \left(1-x\right)}{x}\right]=\lambda h\left(\frac{1}{T}-\frac{1}{T_{m}}\right)$$

where *λ* means the non-ideal nature of the saturated solution, *T_m_* can indicate the melting point of the solute, and *h* is equal to the enthalpy of dissolution per mole of solute divided by the gas constant. In the practical fitting process, the melting point, dissolution temperature, and molar fraction for dehydroabietic acid were used as data. *λ*, *h* were regressed as the parameters for binary and multivariate systems (see Table 5). The equation directly relates the solubility to temperature, avoiding the activity coefficient and greatly simplifying the calculation process.

### 4.4. NRTL Model

The NRTL equation can be applied to both miscible and partially miscible systems [38], and the excess Gibbs free energy for binary systems can be described as Equations (23)–(27):

(23) $$\frac{g^{E}}{RT}=x_{1}x_{2}\left(\frac{\tau_{12}G_{12}}{x_{2}+x_{1}G_{12}}+\frac{\tau_{21}G_{21}}{x_{1}+x_{2}G_{21}}\right)$$

(24) $$G_{12}=exp\left(-\alpha \tau_{12}\right)$$

(25) $$G_{21}=exp\left(-\alpha \tau_{21}\right)\;$$

(26) $$\tau_{12}=\frac{g_{12}-g_{22}}{RT}=\frac{\Delta g_{12}}{RT}$$

(27) $$\tau_{21}=\frac{g_{21}-g_{11}}{RT}=\frac{\Delta g_{21}}{RT}$$

The expression for the solute activity coefficient calculated from the NRTL model can be expressed as [39]:

(28) $$ln\gamma_{1}=x_{2}^{2}\left[\frac{\tau_{21}G_{21}^{2}}{{\left(x_{1}+x_{2}G_{21}\right)}^{2}}+\frac{\tau_{12}G_{12}}{{\left(x_{2}+x_{1}G_{12}\right)}^{2}}\right]$$

(29) $$ln\gamma_{2}=x_{1}^{2}\left[\frac{\tau_{12}G_{12}^{2}}{{\left(x_{2}+x_{1}G_{12}\right)}^{2}}+\frac{\tau_{21}G_{21}}{{\left(x_{1}+x_{2}G_{21}\right)}^{2}}\right]$$

The NRTL equation fitted to the experimental data contains three parameters (Δ*g*_12_, Δ*g*_21_, *α*_12_). In the actual calculation of this experiment (*α*_12_ = 0.3), the NRTL contains only two adjustable parameters. Δ*g*_12_ and Δ*g*_21_ represent two different intermolecular interactions for the Gibbs free energy, and the parameter values are given in Table 6.

### 4.5. UNIQUAC Model

The UNIQUAC equation is a general analog chemical model obtained using the concept of partial composition and statistical thermodynamic methods [40]. It can be used for multivariate mixtures of non-polar and polar components to predict equilibrium data. The model is easy to calculate and applicable to partially miscible systems. Although the model does not accurately describe the data for all systems, it has a wide range of applications in the industry that is well established. For a binary mixture system, the activity coefficient of component i can be represented by the Equation (30):

(30) $$ln\gamma_{i}=ln\frac{\varphi_{i}}{x_{i}}+\frac{z}{2}q_{i}ln\frac{\theta_{i}}{\varphi_{i}}+l_{i}-\frac{\varphi_{i}}{x_{i}}\sum x_{j}l_{j}-q_{i}\left[ln\left(\underset{j}{\sum }\theta_{i}\tau_{ji}\right)-1+\underset{j}{\sum }\frac{\theta_{j}\tau_{ij}}{\sum_{k}\left(\theta_{k}\tau_{kj}\right)}\right]$$

The parameters can be obtained from Equations (31)–(34).

(31) $$\varphi_{i}=x_{i}r_{i}/\underset{j}{\sum }x_{j}r_{j}$$

(32) $$\theta_{i}=x_{i}q_{i}/\underset{j}{\sum }x_{j}q_{j}$$

(33) $$\tau_{ij}=exp\left(-\frac{\Delta u_{ij}}{RT}\right)$$

(34) $$l_{i}=\frac{z}{2}\left(r_{i}-q_{i}\right)-r_{i}+1$$

*z* is the number of interacting molecules in close proximity around the central molecule, taken as *z* = 10. *θ_i_* and $\varphi_{i}$ are the average surface area fraction and average volume fraction of component i, respectively. *r_i_* and *q_i_* are the structural parameters of pure component *i*, which describe the molecular size and surface area, respectively. *r_i_* and *q_i_* can be used to calculate the R_i_ and Q_i_ of each group by the group contribution method. $\Delta u_{12}$ and $\Delta u_{21}$ are the two adjustable parameters of the UNIQUAC equation (see Table 7). The values of *q* and *r* can be calculated by the equations [41]:

(35) $$q_{i}=\frac{\sum_{i}Q_{i}}{A_{vm}}$$

(36) $$r_{i}=\frac{\sum_{i}R_{i}}{V_{vm}}$$

*Q_i_* is the surface area parameter of group *i*, and *R_i_* is the volume parameter of group *i*. *Q_i_* and *R_i_* can be obtained from the van der Waals volume *A_vm_* and surface area *V_vm_*. The results of the calculation of *r* and *q* are shown in the Table S3.

### 4.6. The Modified Wilson Model

Comer and Kopecni proposed the equation Wilson for the correction of solute solubility in mixed solvents, which is simple in structure and has only two correlation parameters for binary mixed solvent systems. The equation has the following form for binary mixed solvent systems [42]:

(37) $$-lnx_{m}=1-\frac{w_{1}\left[1+ln\left(x_{1}\right)\right]}{w_{1}+w_{2}\lambda_{12}}-\frac{w_{2}\left[1+ln\left(x_{2}\right)\right]}{w_{2}+w_{1}\lambda_{21}}$$

where λ_12_ and λ_21_ are the two correlation parameters of the modified Wilson equation with some predictive capability (see Table 9); *x_m_* is the molar fraction of solute; and *w*_1_ and *w*_2_ are the mass fractions of the solvent mixture. This equation is obtained by correlating a portion of the data, which can be used to predict the solubility values of solutes at other compositions and temperatures.

### 4.7. The Modified Wilson–van’t Hoff Model

The van’t Hoff equation was introduced into the Wilson model, which represents an integrated model for fitting solute solubility data in a co-solvent system. The Wilson–van’t Hoff equation can be expressed as:

(38) $$-lnx_{m}=1-\frac{w_{1}\left[1+A_{1}+\frac{B_{1}}{T}\right]}{w_{1}+w_{2}\lambda_{12}}-\frac{w_{2}\left[1+A_{2}+\frac{B_{2}}{T}\right]}{w_{2}+w_{1}\lambda_{21}}$$

*A*_1_, *B*_1_, *A*_2_, and *B*_2_ are the van’t Hoff model’s parameters acquired by fitting the solubility of the solute in the pure solvent at different temperatures. The relevant parameters are listed in Table 10.

### 4.8. COSMO-RS Prediction

COSMOtherm software is based on the COSMO-RS theory used to predict saturated vapor pressure, solubility, vapor-liquid phase diagrams, solid-liquid phase diagrams, etc. The software is based on quantitative calculations to forecast the thermodynamic properties of fluids in the absence of experimental data.

The COSMO-RS analysis was performed using COSMOthermX software (version 2020). The theoretical basis of this software is the COSMO-RS model. The molecular conformation of dehydroabietic acid was first optimized by the DMOL3_PBE_20.ctd program package with the PBE DFT function. Then, the COSMO file of dehydroabietic acid was obtained by the TURBOMOLE2021 package, while that of the solvents were obtained from the COSMO-RS database DNP basis set. From the compound information in these COSMO files, the sigma surfaces of the dehydroabietic acid and the solvents could be shown and transformed into corresponding sigma profiles. Finally, the molecular interaction energies and solubility values were calculated by inputting the above COSMO files into the COSMOthermX software.

The calculation of $\Delta G_{fus}$ must be considered when predicting the solubility of dehydroabietic acid with COSMOtherm. COSMO-RS predicts the solubility mainly from the following equation [43]:

(39) $$\log_{10}x_{i}{}^{sol\left(n+1\right)}=\left[\mu_{i}^{pure}-\mu_{i}^{s}\left(x_{i}^{sol\left(n\right)}\right)-\max\left(0,\Delta G_{fus}\right)\right]/\left(RTln\left(10\right)\right)$$

where *x_i_^sol^* is the mole fraction of solute dissolved in the corresponding solvents. *μ_i_^pure^* and *μ_i_^s^* represent the chemical potential of pure solute and the infinite dilution chemical potential of solute in the corresponding solvents, respectively. The value of $\Delta G_{fus}$reflects the Gibbs free energy of fusion, which significantly affects the precision of the estimated solubility values. The $\Delta G_{fus}$was obtained from the DSC data or the reference data.

## 5. Conclusions

In this paper, the solubility of dehydroabietic acid was investigated in three monosolvents ((−)-α-pinene, (−)-β-caryophyllene, and p-cymene) and three mixed solvents ((−)-α-pinene + p-cymene, (−)-β-caryophyllene + p-cymene, and (−)-α-pinene + (−)-β-caryophyllene). The experimental results show that with increasing temperature, the solubility of dehydroabietic acid in the selected solvents increases. The solubility of dehydroabietic acid in p-cymene is significantly greater than that in (−)-α-pinene and (−)-β-caryophyllene. In addition, the solubility of dehydroabietic acid in binary solvents containing p-cymene is significantly higher than that not containing p-cymene. An analysis of the solubility of dehydroabietic acid with the solvent using the HSP showed that the best miscibility with p-cymene was achieved. The solubility of dehydroabietic acid in selected solvents systems was fitted using the NRTL, UNIQUAC, modified Apelblat, modified Wilson, modified Wilson–van’t Hoff, and λh models; with the modified Apelblat model showing the best correlation with the lowest AIC value for dehydroabietic acid in monosolvents. In addition, the λh equation was the most appropriate to correlate the solubility of dehydroabietic acid in binary solvents.

The solubility of dehydroabietic acid in selected solvents systems was predicted by COSMO-RS. The analysis of the sigma profiles of dehydroabietic acid with solvent molecules, and the intermolecular interactions, showed that the synergetic effect of multiple interaction energies offers a significant contribution to the dissolution process of dehydroabietic acid. Δ_sol_H^0^, Δ_sol_S^0^, and Δ_sol_G^0^ were obtained from the thermodynamic analysis according to the van’t Hoff equation, demonstrating the dissolution is an irreversible heat-absorbing process.

## Figures and Tables

**Table 1:** Values of experimental mole-fraction solubility (*x*_1_*^exp^*) and calculated solubility data (*x*_1_*^cal^*) of dehydroabietic acid in monosolvents ((−)-α-pinene, p-cymene, and (−)-β-caryophyllene) at different temperature T and pressure P = 101.3 kPa ^a^.

T	x_1_^exp^	x_1_^cal^	x_1_^cal^	x_1_^cal^	x_1_^cal^
		Modified Apelblat	λh	NRTL	UNIQUAC
(−)-α-pinene					
299.45	0.09417	0.09402	0.09367	0.09319	0.09447
303.55	0.10171	0.10209	0.10205	0.10167	0.10239
307.38	0.11025	0.11017	0.11036	0.11007	0.11027
311.40	0.11894	0.11926	0.11961	0.11944	0.11920
313.58	0.12536	0.12445	0.12486	0.12474	0.12425
318.00	0.13501	0.13560	0.13604	0.13606	0.13532
322.16	0.14751	0.14690	0.14722	0.14736	0.14652
325.37	0.15607	0.15617	0.15631	0.15656	0.15582
328.40	0.16499	0.16538	0.16526	0.16564	0.16513
330.70	0.17197	0.17270	0.17232	0.17278	0.17257
333.24	0.18171	0.18111	0.18037	0.18094	0.18121
337.83	0.19733	0.19721	0.19565	0.19643	0.19792
p-cymene					
300.60	0.17951	0.17973	0.17807	0.15809	0.17732
305.84	0.18870	0.18845	0.18831	0.17306	0.18767
308.85	0.19381	0.19394	0.19444	0.18217	0.19415
311.54	0.19877	0.19915	0.20008	0.19055	0.20001
314.24	0.20598	0.20468	0.20589	0.19894	0.20510
318.77	0.21406	0.21468	0.21600	0.21427	0.21628
321.54	0.22128	0.22125	0.22243	0.22373	0.22248
325.29	0.23048	0.23072	0.23142	0.23714	0.23175
328.20	0.23874	0.23856	0.23866	0.24780	0.23896
331.08	0.24592	0.24676	0.24604	0.25886	0.24696
334.75	0.25801	0.25786	0.25579	0.27305	0.25670
336.16	0.26287	0.26233	0.25965	0.27863	0.26059
(−)-β-caryophyllene					
301.35	0.09789	0.09837	0.09858	0.09881	0.09980
304.60	0.10524	0.10511	0.10514	0.10534	0.10591
309.34	0.11578	0.11547	0.11529	0.11546	0.11551
313.25	0.12512	0.12450	0.12420	0.12432	0.12402
316.65	0.13314	0.13271	0.13237	0.13244	0.13193
319.75	0.14076	0.14049	0.14016	0.14018	0.13956
323.60	0.15004	0.15054	0.15031	0.15026	0.14966
325.96	0.15626	0.15692	0.15681	0.15669	0.15618
328.53	0.16307	0.16405	0.16413	0.16393	0.16360
331.53	0.17201	0.17263	0.17300	0.17267	0.17266
333.93	0.17997	0.17968	0.18037	0.17990	0.18027
336.86	0.18976	0.18853	0.18969	0.18905	0.19004

^a^ The standard uncertainty of u(T) = 0.1 K, u(p) = 0.2 kPa. The relative standard uncertainty of u_r_(x) = 0.01.

**Table 2:** Hansen solubility parameter (HSP) values for selected solvents ^a^.

Solvents	δ (MPa)^0.5^	$\Delta \delta_{t}(\mathrm{MPa}{)}^{0.5}$
(−)-α-pinene	17.6069	3.1075
p-cymene	18.0516	2.6628
(−)-β-caryophyllene	17.7463	2.9681
p-cymene + (−)-β-caryophyllene	17.8990	2.8154
p-cymene + (−)-α-pinene	17.8293	2.8851
(−)-α-pinene + (−)-β-caryophyllene	17.6766	3.0378

^a^ Taken from Ref. [17].

**Table 3:** Values of experimental mole-fraction solubility (*x*_1_*^exp^*) and calculated solubility data (*x*_1_*^cal^*) of dehydroabietic acid in three binary solvents (p-cymene + (−)-β-caryophyllene, p-cymene + (−)-α-pinene, and (−)-α-pinene + (−)-β-caryophyllene) at different temperature T and pressure P = 101.3 kPa ^a^.

T	x_1_^exp^	x_1_^cal^	x_1_^cal^	x_1_^cal^
		Modified Wilson	Modified Wilson with Van’t Hoff	λh
p-cymene (w_1_ = 0.5) + (−)-β-caryophyllene				
295.54	0.14409	0.14906	0.14021	0.14321
298.90	0.15020	0.15332	0.14817	0.14971
303.24	0.15784	0.15909	0.15768	0.15844
308.34	0.16867	0.16799	0.16926	0.16923
313.28	0.17961	0.17714	0.18089	0.18024
318.45	0.18941	0.18636	0.19349	0.19241
321.90	0.19954	0.19588	0.20214	0.20092
325.75	0.21104	0.20800	0.21202	0.21080
329.26	0.22113	0.21914	0.22123	0.22018
332.24	0.22944	0.22907	0.22920	0.22843
334.43	0.23727	0.23889	0.23515	0.23468
337.75	0.24663	0.25136	0.24431	0.24444
p-cymene (w_1_ = 0.5) + (−)-α-pinene				
297.45	0.14197	0.14562	0.14041	0.14199
301.84	0.15089	0.15577	0.14998	0.15086
303.67	0.15530	0.15588	0.15407	0.15468
309.45	0.16693	0.17006	0.16738	0.16726
313.55	0.17713	0.17577	0.17719	0.17667
318.15	0.18642	0.17897	0.18855	0.18774
320.87	0.19347	0.18762	0.19544	0.19455
324.46	0.20403	0.20123	0.20474	0.20386
327.47	0.21218	0.21115	0.21271	0.21195
330.85	0.22095	0.21567	0.22184	0.22137
334.05	0.23113	0.23967	0.23067	0.23062
338.75	0.24673	0.25128	0.24395	0.24483
(−)-α-pinene (w_1_ = 0.5) + (−)-β-caryophyllene				
295.15	0.08338	0.09389	0.08668	0.08479
300.45	0.09577	0.10151	0.09772	0.09612
303.57	0.10417	0.10704	0.10467	0.10328
307.25	0.11507	0.11477	0.11329	0.11221
313.07	0.12970	0.12619	0.12791	0.12744
315.99	0.13747	0.13280	0.13572	0.13562
319.63	0.14686	0.14134	0.14589	0.14634
323.93	0.15967	0.15405	0.15857	0.15976
327.27	0.16973	0.16499	0.16892	0.17077
331.14	0.18024	0.17739	0.18147	0.18419
335.17	0.19484	0.19648	0.19518	0.19895
339.46	0.20909	0.21751	0.21052	0.21555

^a^ The standard uncertainty of u(T) = 0.1 K, u(p) = 0.2 kPa. The relative standard uncertainty of u_r_(x) = 0.01.

**Table 4:** Regression results and model parameters of the modified Apelblat model of dehydroabietic acid in solvents.

Solvent	A	B	C	10^3^RMSD	100ARD
(−)-α-pinene	−48.47524	521.07863	7.78171	0.49179	0.29696
p-cymene	−111.46958	4249.61692	16.75777	0.53532	0.18554
(−)-β-caryophyllene	9.43067	−2122.40205	−0.82455	0.61823	0.36719

**Table 5:** Regression results and model parameters of the λh model of dehydroabietic acid in solvents.

Solvent	λ	h	10^3^RMSD	100ARD
(−)-α-pinene	0.63102	2847.00662	0.76833	0.41582
p-cymene	0.13981	3301.66787	1.46170	0.50337
(−)-β-caryophyllene	0.55282	3043.30450	0.65581	0.41461
(w_1_ = 0.5) p-cymene + (−)-β-caryophyllene	0.25625	3193.67354	1.48598	0.60355
(w_1_ = 0.5) p-cymene + (−)-α-pinene	0.29405	3117.90536	0.80205	0.29386
(w_1_ = 0.5) (−)-α-pinene + (−)-β-caryophyllene	0.89487	2319.29702	2.81877	1.40869

**Table 6:** Regression results and model parameters of the NRTL model of dehydroabietic acid in solvents.

Solvent	Δg_12_	Δg_21_	10^3^RMSD	100ARD
(−)-α-pinene	4616.96557	−2402.45933	0.67424	0.42365
p-cymene	−4165.94631	5604.35736	12.00745	4.89582
(−)-β-caryophyllene	6012.56622	−2772.68602	0.59964	0.38518

**Table 7:** Regression results and model parameters of the UNIQUAC model of dehydroabietic acid in solvents.

Solvent	Δu_12_	Δu_21_	10^3^RMSD	100ARD
(−)-α-pinene	2789.63759	−1207.90976	0.57663	0.34881
p-cymene	3988.26141	−1829.74505	1.42346	0.58455
(−)-β-caryophyllene	3475.77031	−1200.32787	0.88057	0.56537

**Table 8:** Regression results and model parameters of the modified Wilson model of dehydroabietic acid in solvents.

Solvent	λ_12_	λ_21_	10^3^RMSD	100ARD
(w_1_ = 0.5) p-cymene + β-caryophyllene	−1.34019	−1.86192	2.93829	1.37903
(w_1_ = 0.5) p-cymene + α-pinene	−0.94156	−1.04704	4.73645	2.13388
(w_1_ = 0.5) α-pinene + β-caryophyllene	−1.29291	−1.43891	5.41597	3.56843

**Table 9:** Regression results and model parameters of the van’t Hoff model of dehydroabietic acid in solvents.

Solvent	A	B	10^3^RMSD	100ARD
(−)-α-pinene	4.14712	−1952.44054	0.87934	0.47957
p-cymene	1.88763	−1088.87019	2.10495	0.79972
(−)-β-caryophyllene	3.85311	−1859.74233	0.60195	0.36824

**Table 10:** Regression results and model parameters of the modified Wilson–van’t Hoff model of dehydroabietic acid in solvents.

Solvent	λ_12_	λ_21_	10^3^RMSD	100ARD
(w_1_ = 0.5) p-cymene + (−)-β-caryophyllene	−0.15307	133.51780	2.15187	0.93010
(w_1_ = 0.5) p-cymene + (−)-α-pinene	1.88763	−1088.87019	1.38301	0.60838
(w_1_ = 0.5) (−)-α-pinene + (−)-β-caryophyllene	3.8531	−1859.7423	1.60410	1.15952

**Table 11:** Value of the Akaike Information Criterion (AIC) of the fitting model for dehydroabietic acid in the different solvents.

Models	104RSS ^a^	Parameters	AIC ^b^	e^((AICmin-AICi)/2) c^	Akaike Weight ^d^ (ω_i_)
(−)-α-pinene					
modified Apelblat	0.02902	3	−176.8191	1	0.92964
λh	0.07084	2	−168.1110	0.01285	0.01195
NRTL	0.05455	2	−167.2461	0.00834	0.00775
UNIQUAC	0.03990	2	−170.9995	0.05448	0.05065
p-cymene					
modified Apelblat	0.03439	3	−174.7835	1	0.99998
λh	0.25639	2	−152.6758	1.58256 × 10^−5^	1.58253 × 10^−5^
NRTL	17.30147	2	−98.1335	2.26804 × 10^−17^	2.26799 × 10^−17^
UNIQUAC	0.24315	2	−149.3119	2.94382 × 10^−6^	2.94377 × 10^−6^
(−)-β-caryophyllene					
modified Apelblat	0.04586	3	−171.3277	0.74685	0.34783
λh	0.05161	2	−171.9114	1	0.46574
NRTL	0.04315	2	−170.0605	0.39635	0.18459
UNIQUAC	0.09305	2	−160.8386	0.00394	0.00184
p-cymene + (−)-β-caryophyllene					
Modified Wilson	1.03603	2	−135.9183	0.00028	0.00028
Modified Wilson with van’t Hoff	0.55567	2	−143.3940	0.01176	0.01162
λh	0.26498	2	−152.2803	1	0.98810
p-cymene + (−)-α-pinene					
Modified Wilson	2.69207	2	−124.4592	5.55893 × 10^−10^	5.55090 × 10^−10^
Modified Wilson with van’t Hoff	0.22952	2	−154.0039	0.00145	0.00145
λh	0.07719	2	−167.0801	1	0.99855
(−)-α-pinene + (−)-β-caryophyllene					
Modified Wilson	3.51993	2	−121.2417	1.54771 × 10^−5^	1.48933 × 10^−5^
Modified Wilson with van’t Hoff	0.55567	2	−143.3940	1	0.96228
λh	0.95346	2	−136.9149	0.03918	0.03770

^a^ RSS is the residual sum of squares. ^b^ AIC is the Akaike Information Criterion value for each model. ^c^ AIC_min_ is the minimum value of the compared models, and AICi is the value of the ith model. ^d^ The Akaike weight is the probability of each model within the interval [0, 1] and to the sum of 1.

**Table 12:** Values of apparent thermodynamic properties of solutions (Δ_sol_G^0^, Δ_sol_H^0^, Δ_sol_S^0^) of dehydroabietic acid in solvents (P = 101.3 KPa) ^a^.

Solvents	Intercept	Slope	Δ_sol_G^0^/kJ mol^−1^	Δ_sol_H^0^/kJ mol^−1^	Δ_sol_S^0^/J·mol^−1^K^−1^	δ_H_	δ_TS_
(−)-α-pinene	−1.9770	−1952.4405	5.2401	16.2326	34.4791	0.5962	0.4038
p-cymene	−1.5221	−1088.8702	4.0412	9.05287	15.6937	0.6437	0.3563
(−)-β-caryophyllene	−1.9576	−1859.7423	5.2090	15.4619	32.0347	0.6013	0.3987
(w_1_ = 0.5) p-cymene + (−)-β-caryophyllene	−1.6522	−1277.2243	4.3636	10.6188	19.6920	0.6293	0.3707
(w_1_ = 0.5) p-cymene + (−)-α-pinene	−1.6744	−1328.6663	4.4249	11.0465	20.8319	0.6252	0.3748
(w_1_ = 0.5) (−)-α-pinene + (−)-β-caryophyllene	−1.9661	−2060.5766	5.1924	17.1316	37.5853	0.5893	0.4107

^a^ The standard uncertainty u: u(Δ_sol_G^0^) = 0.05 kJ mol^−1^, u(Δ_sol_H^0^) = 0.05 kJ mol^−1^, u(Δ_sol_S^0^) = 0.05 J mol^−1^K^−1^.

**Table 13:** Values of experimental mole-fraction solubility (x_1_) of dehydroabietic acid in the binary solvent at temperature T and pressure P = 101.3 kPa ^a^.

Mass Fraction (w_1_)	T/K	*x*_1_
(−)-α-pinene(w_1_) + p-cymene		
0.1	304.65	0.18572
0.2	309.45	0.18739
0.3	311.40	0.18672
0.4	313.45	0.18583
0.5	313.55	0.17713
0.6	314.25	0.17325
0.7	313.55	0.15997
0.8	314.95	0.15047
0.9	318.15	0.14841
p-cymene(w_1_) + (−)-β-caryophyllene		
0.1	323.50	0.16368
0.2	316.64	0.15576
0.3	317.95	0.17055
0.4	314.70	0.17427
0.5	313.28	0.17961
0.6	312.05	0.18090
0.7	311.20	0.18503
0.8	312.53	0.19505
0.9	313.30	0.20398
(−)-α-pinene(w_1_) + (−)-β-caryophyllene		
0.1	311.40	0.12665
0.2	312.40	0.12751
0.3	313.30	0.12837
0.4	315.55	0.13447
0.5	319.43	0.14686
0.6	319.75	0.14597
0.7	322.35	0.15191
0.8	324.05	0.15619
0.9	325.85	0.16078

^a^ The standard uncertainty of u(T) = 0.1 K. The relative standard uncertainty of u_r_(x) = 0.01, u_r_(p) = 0.05.

**Table 14:** Descriptions of the materials used in the experiments.

Chemical Name	CASRN	Mass Fraction Purity	Source	Analysis Method
dehydroabietic acid	1740-19-8	0.99 ^a^	Laboratory self-made	gas chromatography (GC)
(−)-α-pinene	7785-26-4	0.98 ^b^	Aladdin Biochemical Co., Ltd.	GC
p-cymene	99-87-6	0.99 ^b^	Adamas Pharmaceuticals, Inc.	GC
(−)-β-caryophyllene	87-44-5	0.99 ^b^	TCI(Shanghai)Development Co., Ltd.	GC

^a^ Preparation of samples in the laboratory followed by determination of their purity using gas chromatography. ^b^ The purity of materials is provided by the supplier.

**Table 15:** The Hansen solubility parameter (HSP) for dehydroabietic acid was calculated using the group contribution method ^a^.

Group	Number	E (J·mol^−1^)	V (cm^3^·mol^−1^)
CH_3_	4	4707	33.5
CH_2_	5	4937.12	16.1
CH	2	3430.88	−1.0
C	2	1464.4	−19.2
COOH	1	27,614.4	28.5
Benzene ring	1	31,923.92	33.4
Ring	2	1046	16.0
$\delta =\frac{\sum_{i}E}{\sum_{i}V}$= 20.7144 MPa^0.5^			

^a^ Taken from Ref. [17].

## Data Availability

The data presented in this study are available to all readers according to “MDPI Research Data Policies”.

## Supplementary Materials

The following supporting information can be downloaded, Table S1: Values of experimental mole-fraction solubility (x1exp), relative deviation (RD) of dehydroabietic acid in monosolvents ((−)-α-pinene, p-cymene, (−)-β-caryophyllene) at temperature T and pressure P = 101.3 kPa; Table S2: Values of experimental mole-fraction solubility (x1exp), relative deviation (RD) of dehydroabietic acid in three binary solvents (p-cymene + (−)-β-caryophyllene, p-cymene + (−)-α-pinene, (−)-α-pinene + (−)-β-caryophyllene) at temperature T and pressure P = 101.3 kPa; Table S3: Values of volume parameter (r) and surface parameter (q) for dehydroabietic acid and selected solvents.

## Sample Availability

Samples of the all compounds are available from the authors.
