# Supplementary material for: Experimental Determination and Computational Prediction of Dehydroabietic Acid Solubility in (−)-α-Pinene + (−)-β-Caryophyllene + P-Cymene System
Source: Molecules. 2022 Feb 11;27(4):1220. doi: 10.3390/molecules27041220 (PMC8875717; doi:10.3390/molecules27041220)
Supplement: Supplementary file 1 [file molecules-27-01220-s001.zip › molecules-1568244-supplementary.pdf]

Supplementary Information (SI)
Experimental Determination and Computational Prediction of Dehydroabietic Acid Solubility in (−)-α-Pinene + (−)-β-Caryophyllene + P-Cymene System

Yanmin Qin[1], Xiaopeng Chen[1], Linlin Wang[1], Xiaojie Wei[1], Weijian Nong[2,3], Xuejuan Wei[1], Jiezhen Liang[1*]

[1]Guangxi Key Laboratory of Petrochemical Resource Processing and Process Intensification Technology, School of Chemistry and Chemical Engineering, Guangxi University, Nanning, 530004, P. R. China

[2]China Academy of science and Technology Development Guangxi Bran-ch, Nanning, 530022, China

[3]Guangxi Sci-Tech Development Forest-like Technology Co. LTD, Nanning, 530022, China

AUTHOR INFORMATION

Corresponding Author

Email: ljztony01@163.com

Fax: +86-771-323-3718   Phone: +86-771-3272702

Author Contrubution

Y. Qin contributed to the work as first authors.

Table S1. Values of experimental mole-fraction solubility (x_1^{exp}), relative deviation (RD) of dehydroabietic acid in monosolvents ((-)-α-pinene, p-cymene, (-)-β-caryophyllene) at temperature T and pressure P=101.3 kPa[a].

100RD	100RD	100RD	100RD
**modified Apelblat**	**λh**	**NRTL**	**UNIQUAC**
	(-)-α-pinene		
-0.15646	-0.53384	1.03671	-0.31800
0.37362	0.32952	0.03797	-0.67209
-0.07143	0.09861	0.16099	-0.01716
0.26475	0.56537	-0.41801	-0.21536
-0.72459	-0.39515	0.49702	0.88767
0.44010	0.76107	-0.77465	-0.23082
-0.41678	-0.19751	0.10084	0.67329
0.06109	0.15118	-0.31628	0.15849
0.23886	0.16641	-0.39063	-0.08733
0.42344	0.20314	-0.47053	-0.34988
-0.33305	-0.73595	0.42381	0.27550
-0.05939	-0.85212	0.45636	-0.30010
	p-cymene		
0.12175	-0.80377	11.93094	1.21749
-0.13218	-0.20438	8.28635	0.54411
0.06598	0.32667	6.00650	-0.17311
0.19084	0.65787	4.13293	-0.62606
-0.62995	-0.04450	3.41605	0.42691
0.28778	0.90788	-0.09830	-1.03508
-0.01550	0.51771	-1.10764	-0.54439
0.10575	0.40885	-2.89075	-0.55239
-0.07410	-0.03560	-3.79481	-0.09342
0.34093	0.04880	-5.26349	-0.42453
-0.05737	-0.85885	-5.82855	0.50960
-0.20438	-1.22562	-5.99351	0.86750
	(-)-β-caryophyllene		
0.48566	0.70037	-0.93611	-1.95310
-0.12518	-0.09454	-0.09918	-0.63716
-0.26620	-0.42123	0.27327	0.23528
-0.49514	-0.73174	0.63597	0.88054
-0.32373	-0.58049	0.52414	0.90681
-0.19459	-0.42806	0.41442	0.85000
0.33146	0.18167	-0.14595	0.25161
0.41930	0.35184	-0.27432	0.05245
0.60040	0.64909	-0.52505	-0.32201
0.35830	0.57700	-0.38315	-0.38064

-0.15963	0.22023	0.03802	-0.16721
-0.64670	-0.03908	0.37256	-0.14760

[a]The standard uncertainty of u(T)=0.1 K, u(p)=0.2 kPa. The relative standard uncertainty of $u_r(x)$=0.01.

Table S2. Values of experimental mole-fraction solubility (x_1^{exp}), relative deviation (RD) of dehydroabietic acid in three binary solvents (p-cymene + (-)-β-caryophyllene, p-cymene + (-)-α-pinene, (-)-α-pinene + (-)-β-caryophyllene) at temperature T and pressure P=101.3 kPa[a].

**100RD**	**100RD**	**100RD**
**Modified Wilson**	**Modified Wilson with van't Hoff**	**λh**
	(w_1=0.5) p-cymene + (-)-β-caryophyllene	
-3.33552	2.76674	-0.60889
-2.03496	1.37237	-0.32743
-0.78260	0.10216	0.38172
0.40479	-0.34915	0.33078
1.39438	-0.70857	0.35336
1.63662	-2.11020	1.58609
1.86849	-1.28788	0.69211
1.46154	-0.46312	-0.11388
0.90810	-0.04653	-0.43048
0.16152	0.10260	-0.43901
-0.67814	0.90099	-1.09267
-1.88176	0.95093	-0.88617
	(w_1=0.5) p-cymene + (-)-α-pinene	
-2.50652	1.10918	0.01716
-3.13282	0.60896	-0.01816
-0.37208	0.80046	-0.39638
-1.84053	-0.27000	0.19860
0.77374	-0.03404	-0.25918
4.16271	-1.13016	0.70743
3.11800	-1.01019	0.55814
1.39144	-0.34737	-0.08479
0.48780	-0.24866	-0.10812
2.44818	-0.40230	0.18950
-3.56203	0.19949	-0.22028
-1.81073	1.13978	-0.76860
	(w_1=0.5) (-)-α-pinene + (-)-β-caryophyllene	
-11.19146	-3.95414	1.69368
-5.65101	-2.03802	0.36351
-2.68315	-0.47849	-0.85465
0.26277	1.55016	-2.49076
2.78125	1.38143	-1.74195
3.51297	1.27414	-1.34131
3.90226	0.65487	-0.35211

3.64907	0.68792	0.05528
2.87286	0.47764	0.61282
1.60598	-0.68412	2.19468
-0.83566	-0.17845	2.10734
-3.87273	-0.68628	3.08570

[a]The standard uncertainty of u(T)=0.1 K, u(p)=0.2 kPa. The relative standard uncertainty of $u_r(x)$=0.01.

Table S3. Values of volume parameter (r) and surface parameter (q) for dehydroabietic acid and selected solvents[a].

Chemical name	r	q
dehydroabietic acid	12.300	9.338
(-)-α-pinene	6.0540	4.759
p-cymene	6.0058	4.612
(-)-β-caryophyllene	9.1945	7.367

[a] Taken from Ref[1].

References

[1] Y.J. Zhao, J.L. Si, L. Xia, S.G. Xiang. A fast method for calculating molecular volume and surface area based on elements and chemical bonds. *Comput, Appl. Chem*. **2013**, 30, 739-742.
